# Inflammatory Markers Change with Age, but do not Fall Beyond Reported Normal Ranges

**DOI:** 10.1007/s00005-015-0357-7

**Published:** 2015-08-18

**Authors:** Aleksandra Wyczalkowska-Tomasik, Bozena Czarkowska-Paczek, Magdalena Zielenkiewicz, Leszek Paczek

**Affiliations:** Department of Immunology, Transplantology, and Internal Diseases, Medical University of Warsaw, Warsaw, Poland; Department of Clinical Nursing, Medical University of Warsaw, E. Ciolka 27, 01-445 Warsaw, Poland; Faculty of Mathematics, Informatics and Mechanics, University of Warsaw, Warsaw, Poland

**Keywords:** CRP, Cytokines, Healthy aging, Inflammation

## Abstract

We examined the serum levels of IL-6, IL-8, TNF, IL-6R, TNF-R1, and CRP and the dynamics of changes in these levels according to age. The study included healthy individuals of 20–90 years of age. Participants were divided into subgroups based on their decade of life, and into subgroups of ≥65 or <65 years. Serum cytokine levels were assayed by ELISA, and CRP using an immunoturbidimetric method. Serum CRP levels were within the normal range for all subgroups. The 60- to 70-year age group showed higher CRP than the 20- to 30- (*p* = 0.003), 30- to 40- (*p* = 0.009), and 40- to 50- (*p* = 0.030) year age groups. Serum cytokine levels were low. It was greater in the 60- to 70-year age group than in the 20- to 30- (*p* = 0.008) and 30- to 40- (*p* = 0.040) year groups, and was greater in the 70- to 90-year group than the 20- to 30-year group (*p* = 0.043). Serum TNF-R1 level in the 70- to 90-year group was greater than in all other age groups (*p* = 0.000 for all comparisons). Other measured parameters did not differ between groups. Serum levels of IL-6, CRP, and TNF-R1 were greater in participants ≥65 than <65 years of age. Healthy older people showed low serum levels of CRP and pro-inflammatory cytokines, but higher than in younger population. Therefore, the adjustment of normal ranges in the elderly should be considered. Serum levels of pro-inflammatory cytokines elevated beyond normal ranges indicate particular diseases.

## Introduction

Some studies show aging to be associated with a state of chronic low-grade inflammation and increased serum levels of inflammatory markers, including interleukin (IL)-6, C-reactive protein (CRP), and tumor necrosis factor (TNF) process that has been referred to as “inflammaging” (Michaud et al. 2013). Despite many clinical trials, the mechanism behind these increases has not yet been fully understood. However, it is proposed that the higher inflammatory marker levels are related to increased volume of adipose tissue (especially, visceral), decline of sex hormones, and increased oxidative damage—all of which are common in elderly individuals (Singh and Newman 2011). A chronic state of inflammation may have detrimental consequences, and thus the cause of inflammaging is of great diagnostic and therapeutic importance.

Increased levels of inflammatory markers are generally associated with age-related diseases, including cardiovascular diseases, increased insulin resistance, and cancer (Reuter et al. 2010; Singh and Newman 2011). Low-grade inflammation is also involved in the mechanism underlying age-related sarcopenia, neurodegenerative disorders, and cognitive deficit (Michaud et al. 2013; Trollor et al. 2012). IL-6 and CRP are both associated with mortality risk, and IL-6 and TNF are both markers of frailty (Michaud et al. 2013; Varadhan et al. 2014). It remains unclear whether increased serum levels of particular cytokines are direct causes of adverse effects and of the above-mentioned diseases or, conversely, are the result of existing diseases.

The present study aimed to assess the serum levels of pro-inflammatory cytokines (IL-6, IL-8, and TNF), the IL-6 and TNF receptors (IL-6R and TNF-R1, respectively), and CRP among healthy individuals aged 20–90 years, and to trace the dynamics of changes in these concentrations according to age in the absence of any co-morbidities.

## Materials and Methods

The study included a total of 180 healthy individuals between 20 and 90 years of age (mean age 49 ± 18 years), recruited from their neighborhood, workplaces, nursing homes, and retirement homes. These participants included 94 women and 86 men. None had any chronic diseases in their medical history (including diabetes), and none of the study participants took any medications. All results of standard biochemical analysis were within the reference value ranges, and all participants showed normal blood pressure and BMI values. Table 1 presents the characteristics of the study participants. Every person enrolled into the study gave their written and informed consent to participate, and the study was approved by the Bioethics Committee of the Medical University of Warsaw (No. KB/10/2010).

**Table 1:** Characteristics of study participants stratified by age

Biomarkers (units)	Under 65 years of age (mean ± SD)	Above 65 years of age (mean ± SD)
Weight (kg)	74.72 ± 16.24	73.51 ± 11.29
BMI	24.97 ± 3.94	26.30 ± 2.87
RBC (T/L)	4.82 ± 0.39	4.75 ± 0.40
HGB (g/L)	143.39 ± 12.65	141.46 ± 12.83
HCT (L/L)	0.43 ± 0.03	0.43 ± 0.03
WBC (G/L)	6.16 ± 1.43	6.50 ± 1.48
PLT (G/L)	252.87 ± 56.30	230.85 ± 58.42
Bilirubin (mg/dL)	0.68 ± 0.34	0.65 ± 0.20
Creatinine (mg/L)	0.92 ± 0.17	1.04 ± 0.28
eGFR CKD-EPI (mL/min)	90.16 ± 16.36	64.94 ± 15.18
Cystatin C (mg/L)	0.66 ± 0.10	0.81 ± 0.19
Urine pH	5.84 ± 0.64	5.81 ± 0.69
Glucose (mg/dL)	89.23 ± 10.35	97.44 ± 13.44
ALT (U/L)	28.33 ± 17.86	21.33 ± 7.65
AST	21.30 ± 8.09	22.03 ± 5.29
Cholesterol (mg/dL)	215.85 ± 44.87	201.56 ± 46.44

eGFR CKD-EPI (estimated glomerular filtration rate—according to CKD-EPI formula)

*BMI* body mass index, *RBC* red blood cells, *HGB* hemoglobin, *HCT* hematocrit, *WBC* white blood cells, *PLT* platelets, *ALT* alanine transaminase, *AST* aspartate transaminase

To analyze major laboratory parameters, whole blood samples (2 mL) were drawn in polypropylene tubes and allowed to clot for 30 min before centrifugation. Serum was obtained by centrifugation for 10 min at approximately 1000×*g*. The samples were stored at −80 °C prior to analysis.

Serum cytokine concentrations were determined by enzyme-linked immunosorbent assays (ELISA) using kits from R&D Systems (Minneapolis, Minnesota, USA). Absorbance readings at 450 and 540 nm were taken using a camera to read a spectrophotometric microplate (Biotek Power Wave XS). CRP was measured via an immunoturbidimetric assay that uses reinforced latex particles (CRPLX C-reactive Protein Latex kit; Roche Diagnostics, Indianapolis, Indiana, USA) and a Cobas turbidimetric analyzer. To assess kidney function, serum levels of cystatin C were determined using a particle-enhanced immunonephelometric diagnostic assay (Siemens N Latex Cystatin C; Siemens, Erlangen, Germany). The test was performed using a BN ProSpec nephelometer (Siemens, Erlangen, Germany) following the manufacturer’s protocol.

### Statistical Analysis

Mean values in the individual age groups were compared using analysis of variance (ANOVA) methods, specifically Tukey’s HSD test. The mean values for the two age groups (≥65 and <65 years) were compared using Tukey’s HSD test and Welch’s *t* test. For each investigated parameter, both tests gave the same results.

The correlations between pairs of parameters were studied using linear regression analysis. A linear model was fitted for each pair of parameters, on which two statistical tests of independence were performed: the Student’s *t* test and the* F* test. The null hypothesis that the variables were independent was rejected if the *p* value was <0.05 for each test. The results of the two tests always agreed. All computations were performed in *R*.

## Results

To trace the dynamics of changes in serum levels of the investigated cytokines with age, we divided the participants into subgroups based on the decade of life (Table 2).

**Table 2:** Serum levels of CRP, IL-6, IL-6R, IL-8, TNF, and TNF R1 stratified by age

Parameter (units)	20–30 years mean ± SD	30–40 years mean ± SD	40–50 years mean ± SD	50–60 years mean ± SD	60–70 years mean ± SD	70–90 years mean ± SD
CRP (mg/L)	1.24 ± 1.18 *n* = 30	1.40 ± 0.86 *n* = 30	1.65 ± 1.45 *n* = 30	2.17 ± 1.91 *n* = 30	3.17 ± 3.47 *n* = 30	2.65 ± 1.92 *n* = 30
IL-6 (pg/mL)	0.11 ± 0.59 *n* = 30	0.48 ± 1.49 *n* = 30	0.26 ± 0.98 *n* = 30	0.82 ± 1.66 *n* = 29	2.63 ± 5.73 *n* = 27	2.18 ± 2.83 *n* = 30
IL-6R (ng/mL)	36.93 ± 11.79 *n* = 26	41.15 ± 10.53 *n* = 26	41.11 ± 11.02 *n* = 30	38.87 ± 10.31 *n* = 25	40.74 ± 9.86 *n* = 27	43.04 ± 13.38 *n* = 26
IL-8 (pg/mL)	25.37 ± 53.82 *n* = 30	27.66 ± 97.65 *n* = 29	33.92 ± 72.61 *n* = 30	41.33 ± 72.01 *n* = 29	32.34 ± 95.49 *n* = 29	25.46 ± 45.05 *n* = 30
TNF (pg/mL)	0.00 ± 0.00 *n* = 30	0.57 ± 3.03 *n* = 28	1.84 ± 9.38 *n* = 26	0.65 ± 3.38 *n* = 27	0.80 ± 4.25 *n* = 28	2.44 ± 8.78 *n* = 27
TNF-R1 (ng/mL)	1.19 ± 0.33 *n* = 30	1.19 ± 0.26 *n* = 29	1.19 ± 0.33 *n* = 30	1.33 ± 0.43 *n* = 29	1.49 ± 0.51 *n* = 29	2.02 ± 0.62 *n* = 29

Mean CRP levels in the 60- to 70-year age group was significantly greater compared to the 20- to 30- (*p* = 0.003), 30- to 40- (*p* = 0.009), and 40- to 50- (*p* = 0.030) year age groups. Mean IL-6 levels in the 60- to 70-year age group was significantly greater compared to the 20- to 30- (*p* = 0.008) and 30- to 40- (*p* = 0.040) year age groups; it also significantly differed between the 70- to 90- and 20- to 30-year age groups (*p* = 0.043). The mean TNF-R1 concentration for the 70- to 90-year age group was significantly greater compared to all other age groups (*p* = 0.000 for all comparisons). The levels of IL-6R, IL-8, and TNF showed no significant differences between age groups

Several parameters significantly differed between age groups. The 60- to 70-year age group showed significantly greater mean CRP levels compared to the 20- to 30- (*p* = 0.003), 30- to 40- (*p* = 0.009), and 40- to 50- (*p* = 0.030) year age groups. The 60- to 70-year age group also showed significantly greater mean IL-6 levels compared to the 20- to 30- (*p* = 0.008) and 30- to 40- (*p* = 0.040) year age groups. IL-6 levels also significantly differed between the 70- to 90- and 20- to 30-year age groups (*p* = 0.043). The mean TNF-R1 concentration for the 70- to 90-year age group was significantly greater compared to all other age groups (*p* = 0.000 for all comparisons). The levels of IL-6R, IL-8, and TNF showed no significant differences between age groups.

To detect potential differences in serum levels of the investigated cytokines between the young and elderly, we also divided the participants into two subgroups of ≥65 years of age and <65 years of age (Table 3).

**Table 3:** Serum levels of CRP, IL-6, Il-6R. IL-8, TNF, and TNF-R1 among participants ≥65 or <65 years of age

Parameter (units) (mean ± SD)	Age <65 years of age	Age ≥65 years of age	*p*
CRP (mg/L)	1.89 ± 2.11 *n* = 141	2.62 ± 1.86 *n* = 39	0.0179
IL-6 (pg/mL)	0.83 ± 2.86 *n* = 138	1.87 ± 2.73 *n* = 38	0.0120
IL-6R (ng/mL)	39.7 ± 10.56 *n* = 126	42.7 ± 13.2 *n* = 34	NS
IL-8 (pg/mL)	33.83 ± 81.81 *n* = 138	21.59 ± 40.78 *n* = 39	NS
TNF (pg/mL)	0.79 ± 5.02 *n* = 131	1.73 ± 7.47 *n* = 35	NS
TNF-R1 (ng/mL)	1.25 ± 0.38 *n* = 139	1.98 ± 0.61 *n* = 37	0.000005

*NS* non significant

Table 4 presents the correlation between the investigated cytokines and the serum level of cystatin.

**Table 4:** Correlations between the investigated cytokines and kidney function estimated by cystatin level with the measured concentrations of CRP, IL-6, IL-6R, IL-8, TNF, and TNF-R1

	CRP (mg/L)	IL-6 (pg/mL)	IL-6R (ng/mL)	IL-8 (pg/mL)	TNF (pg/mL)	TNF-R1 (ng/mL)
Age (years)	*p* = 0.0000 *R* = 0.3105	*p* = 0.00009 *R* = 0.2869	NS	NS	NS	*p* = 0.0000 *R* = 0.5258
Serum cystatin (mg/mL)	*p* = 0.0105 *R* = 0.1936	*p* = 0.0027 *R* = 0.2280	*p* = 0.0160 *R* = 0.1926	NS	NS	*p* = 0.0000 *R* = 0.4965
CRP (mg/L)		*p* = 0.0008 *R* = 0.2494	NS	NS	NS	*p* = 0.0044 *R* = 0.2135
IL-6 (pg/mL)	*p* = 0.0008 *R* = 0.2494		NS	*p* = 0.0000 *R* = 0.3734	NS	*p* = 0.0001 *R* = 0.2900
IL-6 R (ng/mL)	NS	NS		NS	NS	*p* = 0.0072 *R* = 0.2124
IL-8 (pg/mL)	NS	*p* = 0.0000 *R* = 0.3734	NS		NS	NS
TNF (pg/mL)	NS	NS	NS	NS		NS
TNF-R1 (ng/mL)	*p* = 0.004 *R* = 0.2135	*p* = 0.0001 *R* = 0.2900	*p* = 0.0072 *R* = 0.2124	NS	NS	

*NS* non significant

## Discussion

Immune system function generally declines with aging and is called immunosenescence. Immunosenescence is based upon three theories: the autoimmune theory based on the decreased ability to recognize between invaders and normal tissues, the immune deficiency theory based on the diminished effectiveness of the immune system, and the immune dysregulation theory based on the disruption of the regulation between multiple components of the immune system. Immunosenescence is a complex process, accompanied by some changes, for instance compromised function in several cells including lymphocytes (naïve, effector, and memory), regulatory T and B cells, monocytes, neutrophils, and NK cells, as reflected by increased susceptibility to infectious diseases, altered response to vaccination, increased prevalence of particular diseases, and chronic pro-inflammatory states connected with non-communicable diseases—including cardiovascular and neurodegenerative diseases, diabetes, declining cognitive function, and frailty. Immunosenescence affects individuals to a different extent, mainly because the dysfunction of the immune system is influenced by genetic and environmental factors. Inflammatory response is an innate defense, which is less affected by age than adaptive response. (Bueno et al. 2014; Castelo-Branco and Soveral 2014; Franceschi et al. 2000; Yao and Moorman 2013). Many investigators have reported that aging is associated with increased levels of pro-inflammatory cytokines, including IL-6, TNF, CRP, and TNF-R1 (Singh and Newman 2011; Varadhan et al. 2014). These cytokines are involved in the pathogenesis of many diseases, mainly cardiovascular diseases, diabetes, and atherosclerosis (Castelo-Branco and Soveral 2014). These cytokines are also predictors of mortality, with low inflammatory marker levels associated with better survival (Giovannini et al. 2011). However, it remains unclear whether the above-mentioned non-communicable diseases are the cause of pro-inflammatory state or are the reason of it.

In the present study, we assessed the serum levels of CRP and particular cytokines in healthy aging.

First, we analyzed the serum levels of CRP, IL-6, IL-8, TNF, IL-6R, and TNF-R1 in healthy individuals aged 20–90 years, stratified by different decades of life. We then analyzed these data into two subgroups: participants ≥65 and <65 years of age. With advanced age, we detected slight increases of CRP and some cytokines. We also confirmed the increases of IL-6, CRP, and TNF-R1 in healthy individuals aged over 65 years compared to younger participants. No statistically significant difference was found in TNF levels, but there was a clear trend of increase with age. However, despite the observed increases with age, serum levels of the investigated cytokines were generally low in all age groups. There are currently no clinical thresholds for cytokines, including IL-6, but the Whitehall II study proposed that an IL-6 serum level of >2 ng/L is considered high (Akbaraly et al. 2013). In our present group of participants above 65 years of age, the average IL-6 serum level was below this limit. The higher age group also had serum CRP levels within the normal range of 0.2–3.0 mg/L according to the current edition of Harrisson’s Principles of Internal Medicine (Kratz et al. 2012).

The presently obtained results do not contradict the previous findings, indicating that chronic inflammatory states contribute to disabilities and diseases of aging (Guarner and Rubio-Ruiz 2014; Singh and Newman 2011). Many people above 65 years of age suffer from various diseases, with an over 70 % prevalence of multiple chronic conditions in elderly populations (Bahler et al. 2015). The group enrolled in the present study comprised a smaller proportion of disease-free seniors. Age is an independent risk factor for metabolic syndrome, diabetes, and cardiovascular diseases (Tereshina 2009). Multiple morbidities could share common mechanisms, such as telomere shortening, activation of mTOR signaling, impaired autophagy, mitochondrial dysfunction, stem cell exhaustion, abnormal miRNA profiles, or epigenetic changes. Immunosenescence also belongs to this milieu (Barnes 2015). It is also possible that a particular abnormality could trigger the production of pro-inflammatory cytokines, which could then act as an independent risk factor for other diseases (Ferrucci et al. 2005). The results of our investigation conducted in healthy seniors do not clearly confirm the inflammatory state; however, the need to adjust “normal ranges” of CRP and pro-inflammatory cytokines for the elderly population should be considered. The population differences should also be taken into account, due to regional differences in cytokine genotype and polymorphism (Capurso et al. 2004). Also, diet is believed to have a major influence on inflammatory signaling. It could regulate the metabolic pathways and bioenergetics that can be translated into stable epigenetic pattern of gene expression (Szarc vel Szic et al. 2015).

Our results are in line with the results reported by Ferrucci et al. (2005), who stated that at least part of the “pro-inflammatory state” in older persons is related to the associated high prevalence of cardiovascular risk factors and morbidity—not to age exclusively and with those reported by Akbaraly et al. (2013). They found that only high serum IL-6 levels (e.g., ≥2.0 ng/L) halved the odds of successful aging (free of major chronic diseases and with optimal physical, mental, and cognitive function) compared with lower IL-6 levels. These results were unchanged when the IL-6 levels were replaced with CRP levels in regression analysis, or when obese participants were excluded from analysis. Conversely, low serum levels of IL-6 and CRP were connected with successful aging (Akbaraly et al. 2013).

Our present results showed relatively high standard deviations, indicating large inter-individuals differences. There may be complex reasons for the observed slight increases of particular cytokines. In former centuries, the human lifespan averaged 50 years, while it is now 80–100 years. This longer lifespan is associated with a greater antigenic burden and greater lifelong antigenic stress associated with continuous and unavoidable exposure to various antigens, viruses, and bacteria, as well as food and self-molecules (Franceschi et al. 2000). This situation could lead to a well-known reduction of naïve T cells and accumulation of effector T and memory cells, as well as up-regulation of innate immunological defense, connected with the slight increase in pro-inflammatory cytokines (Castelo-Branco and Soveral 2014). The increase in serum levels of pro-inflammatory cytokines and CRP could also result from the decline in kidney function that is observed with age. Supporting this theory, we found that CRP, IL-6, IL-6R, and TNF-R1 levels were significantly positively correlated with serum levels of the renal function marker cystatin (Carbonnel et al. 2008).

Interestingly, the serum levels of IL-6, IL-8, and CRP tended to decrease in the subgroup of 70–90 years of age; however, the levels in this group did not significantly differ from those in the subgroup aged 60–70 years. Inflammatory cytokines and chemokines—including IL-6, IL-8, and TNF—are products of the classical pro-inflammatory TLR signaling pathway (Frazão et al. 2013). According to the results obtained by van Duin et al. (2007), older adults have impaired responses to monocyte TLR1/2-specific stimulation, with decreased TNF, IL-6, and IL-8 production observed in older adults when compared with younger ones.

Our results also showed that the patterns of changes with aging differed among the various cytokine serum levels. IL-6 increased slightly with age, while IL-8 did not. This is in line with previously reported results. IL-8 is one of the most important chemokines responsible for recruiting circulating neutrophils to a site of infection, e.g., chemotaxis, which is probably not affected by age (Castelo-Branco and Soveral 2014; Franceschi et al. 2000). However, some data indicate that aging is accompanied by reduced expression of Toll-like receptor (TLR)1 in polymorphonuclear leukocytes. Qian et al. (2014) reported that in older adults, stimulation through TLR1 leads to lower IL-8 production.

In conclusion, here we show that healthy elderly individuals show low serum levels of CRP and pro-inflammatory cytokines, but higher when compared to the younger population. Therefore, the definition of normal ranges in the elderly should be considered. Elevated serum levels of pro-inflammatory cytokines above normal ranges indicate the coexistence of diagnosed or undiagnosed disease.

## References

[CR1] Akbaraly TN, Hamer M, Ferrie J (2013). Chronic inflammation as determinant of future aging phenotypes. CMAJ.

[CR2] Bahler C, Huber CA, Brungger B (2015). Multimorbidity, health care utilization and costs in an elderly community-dwelling population: a claim data based observational study. BMC Health Serv Res.

[CR3] Barnes PJ (2015). Mechanisms of development of multimorbidity in the elderly. Eur Respir J.

[CR4] Bueno V, Sant’Anna OA, Lord JM (2014). Ageing and myeloid-derived suppressor cells: possible involvement in immunosenescence and age-related disease. Age.

[CR5] Capurso C, Solfrizzi V, D’Introno A (2004). Interleukin 6-174 G/C promoter gene polymorphism and sporadic Alzheimer’s disease: geographic allele and genotype variations in Europe. Exp Gerontol.

[CR6] Carbonnel C, Seux V, Pauly V (2008). Estimation of the glomerular filtration rate in elderly inpatients: comparison of four methods. Rev Med Interne.

[CR7] Castelo-Branco C, Soveral I (2014). The immune system and aging: a review. Gynecol Endocrinol.

[CR8] Ferrucci L, Corsi A, Lauretani F (2005). The origins of age-related proinflammatory state. Blood.

[CR9] Franceschi C, Bonafe M, Valensin S (2000). Human immunosenescence: the prevailing of innate immunity, the failing of clonotyping immunity, and the filling of immunological space. Vaccine.

[CR10] Frazão JB, Errante PR, Condino-Neto A (2013). Toll-like receptors’ pathway disturbances are associated with increased susceptibility to infections in humans. Arch Immunol Ther Exp.

[CR11] Giovannini S, Onder G, Liperoti R (2011). Interleukin-6, C-reactive protein, and tumor necrosis factor-alpha as predictors of mortality in frail, community-dwelling elderly individuals. J Am Geriatr Soc.

[CR12] Guarner V, Rubio-Ruiz ME (2014). Low-grade systemic inflammation connects aging, metabolic syndrome and cardiovascular disease. Interdiscip Top Gerontol.

[CR13] Kratz A, Pesce MA, Basner RC, Longo DL, Fauci AS, Kasper DL (2012). Appendix: laboratory values of clinical importance. Harrison’ s principles of internal medicine.

[CR14] Michaud M, Balardy L, Moulis G (2013). Proinflammatory cytokines, aging, and age-related diseases. J Am Med Dir Assoc.

[CR15] Qian F, Guo X, Wang X (2014). Reduced bioenergetics and toll-like receptor 1 function in human polymorphonuclear leukocytes in aging. Aging.

[CR16] Reuter S, Gupta SC, Chaturvedi MM (2010). Oxidative stress, inflammation, and cancer: how are they linked?. Free Radic Biol Med.

[CR17] Singh T, Newman AB (2011). Inflammatory markers in population studies of aging. Aging Res Rev.

[CR18] Szarc vel Szic KS, Declerc K, Vidakovic M (2015). From inflammaging to healthy aging by dietary lifestyle choices: is epigenetics the key to personalized nutrition?. Clin Epigenetics.

[CR19] Tereshina EV (2009). Metabolic abnormalities as a basis for age-dependent diseases and aging? State of the art (Article in Russian). Adv Gerontol.

[CR20] Trollor JN, Smith E, Agars E (2012). The association between systemic inflammation and cognitive performance in the elderly: the Sydney memory and Ahing Study. Age.

[CR21] van Duin D, Mohanty S, Thomas V, Ginter S, Montgomery RR, Fikrig E, Allore HG, Medzhitov R, Shaw AC (2007). Age-associated defect in human TLR-1/2 function. J Immunol.

[CR22] Varadhan R, Yao W, Matteini A (2014). Simple biologically informed inflammatory index of two serum cytokines predicts 10 year all-cause mortality in older adults. J Gerontol A Biol Sci Med Sci.

[CR23] Yao ZQ, Moorman JP (2013). Immune exhaustion and immune senescence: two distinct pathways for HBV vaccine failure during HCV and/or HIV infection. Arch Immunol Ther Exp.

